# Idiopathic Parkinson’s disease phenotype related to *C9ORF72* repeat expansions: contribution of the neuropsychological assessment

**DOI:** 10.1186/1756-0500-6-343

**Published:** 2013-08-29

**Authors:** Mariam Annan, Émilie Beaufils, Ursule-Catherine Viola, Patrick Vourc’h, Caroline Hommet, Karl Mondon

**Affiliations:** 1 Department of neurology, University hospital center of Tours, 2 bd Tonnellé, 37044 Tours, France; 2 University of Tours, France; 3 Memory Clinic, University hospital center of Tours, 2 bd Tonnellé, 37044 Tours, France; 4 Department of geriatrics, University hospital center of Tours, 2 bd Tonnellé, 37044 Tours, France; 5 INSERM U930, Tours, France; 6 Department of biochemistry and molecular biology, University hospital center of Tours, 2 bd Tonnellé, 37044 Tours, France

**Keywords:** *C9ORF72* repeat expansion, Parkinson’s disease, Cognition

## Abstract

**Background:**

Expanded GGGGCC hexanucleotide repeats in the non-coding region of the *C9ORF72* gene was recently identified as being responsible for over 40% of the cases of amyotrophic lateral sclerosis associated with frontotemporal lobar degeneration, in various extrapyramidal syndromes including supranuclear gaze palsy and corticobasal degeneration, and in addition, has been found to be a rare genetic cause of isolated Parkinsonism. To our knowledge, there is no published data concerning the neuropsychological evaluation of patients diagnosed with idiopathic Parkinson’s disease related with *C9ORF72* repeat expansions.

**Case presentation:**

We report the results of the comprehensive neuropsychological evaluation in a newly described case in the literature (the sixth) of a patient presenting isolated idiopathic Parkinson’s disease associated with *C9ORF72* repeat expansions.

The decrease in the patient’s prefrontal functions resulted in a slight decrease in global efficiency. These abnormalities did not appear to be different, with respect to the deficit observed and the intensity of the cognitive impairment, from those classically observed in cases of sporadic idiopathic Parkinson’s disease. Our patient also exhibited a significant impairment in visual gnosis.

**Conclusions:**

If confirmed in other patients, visuoperceptive deficits in idiopathic Parkinson’s disease could represent a red flag that should prompt the clinician to perform addition diagnostic procedures. A thorough neuropsychological assessment may prove to be useful for detecting idiopathic Parkinson’s disease in patients who are suspected of having repeat abnormalities of *C9ORF72* expansions.

## Background

Expanded GGGGCC hexanucleotide repeats in non-coding regions of the *C9ORF72* gene was recently identified as being responsible of over 40% of the cases of amyotrophic lateral sclerosis associated with frontotemporal lobar degeneration [1,2].

Recent publications have shown involvement of *C9ORF72* repeat expansions in various extrapyramidal syndromes including supranuclear gaze palsy and corticobasal degeneration [3] and has also be found to constitute a rare genetic cause of isolated parkinsonism [4] in some patients who fulfil the UK Parkinson’s Disease Society Brain Bank [5] criteria for idiopathic Parkinson’s disease (IPD).

To our knowledge, there is no published data on the contribution of the neuropsychological assessment in these last-mentioned patients.

We report the results of the thorough neuropsychological assessment of a newly described case in the literature (the sixth) of a patient presenting isolated IPD associated with *C9ORF72* repeat expansions.

## Case presentation

A 63 year-old woman was referred to our department because of a strong family history of neurological diseases: her mother had died at the age of 59 years with Alzheimer’s dementia and her two sisters had died at age 69 and 59 y respectively with a diagnosis of frontotemporal lobar degeneration associated with minor symptoms of parkinsonism. A *C9ORF72* repeat expansion was found by genetic analysis in the youngest sister.

Her personal medical history included appendectomy, diabetes mellitus, and a complete excision of a melanoma.

Parkinsonism was first diagnosed in 2009 at the age of 63 years when left akinesia and tremor appeared. Her symptoms responded poorly to levodopa, and the response rate was estimated to be approximately 20% by both the patient and her husband, and progressively worsened. At the onset, neurological examination revealed a resting tremor associated with akinesia. The Parkinsonism was bilateral but was clearly predominant on the left side. Rapid and alternative movements in the left hand were severely hypometric. Her left lower extremity was also akinetic, and she had severe difficulties in performing repetitive movements. She had no limitation in oculomotor movements and no gait disorder. She never developed motor fluctuations or dyskinesias. The patient fulfilled the criteria for IPD according to the UK Parkinson’s Disease Society Brain Bank [5]: i/ the parkinson’s syndrome was defined by bradykinesia, with a 4–6 Hz resting tremor associated with muscular rigidity; ii/ no exclusion criteria were observed; iii/ the clinical presentation included more than 3 supportive prospective criteria (unilateral onset, rest tremor, progressive clinical course, persistent asymmetry affecting the side most affected at the onset of the disease).

A complete neuropsychological assessment two years after the onset of the disease (2011) revealed preservation of global cognitive efficiency (MATTIS DRS score: 137/144 [6]), difficulties in the executive functions (with a score at 10/18 on the Frontal Assessment Battery [7]), no difficulty in mental flexibility (Trail making test [8]) or sensitivity to interference (stroop test [9]), and global slowness (Coding subtest of the WAIS [10]).

After the discovery of the disease, we asked the patient for permission to test for the *C9ORF72* gene abnormality. The number of repeat expansions was superior to 30 and was therefore considered to be abnormal.

In 2012, a comprehensive neuropsychological assessment was performed (see results in Table 1) and revealed: a slight decrease in global cognitive efficiency (MATTIS DRS score [6]), normal efficiency in long-term verbal episodic (free and cued recall test [11]) and visual (modified Taylor complex figure [12]) memories, language (oral denomination [13]), praxis (Mahieux’s battery [14] and visuospatial skills (Modified Taylor complex figure - copy [12]); low scores in short-term memory (WAIS-III digit span subtest [10]), and the prefrontal functions including verbal initiation (verbal fluencies [15]), and conflicting task (stroop test [9]). Unfortunately, our patient also exhibited significant difficulties in visual gnosis (Poppelreuter [16] and PEGV [17]).

**Table 1:** Results of the neuropsychological assessment

**Cognitive domain / Neuropsychological test (Range)**		Score*
Global efficiency		
	MATTIS DRS (0–144)	**133**
Memory		
	Free and cued recall test	
	Immediate recall (0–16)	**13**
	Free recall (0–48)	25
	Total recall (0–48)	44
	% sensitivity (0–100)	83%
	WAIS-R digit span subtest (direct)	**4**
	WAIS-R digit span subtest (reverse)	3
	Modified Taylor Complex figure (memory) (0–36)	17
Language		
	DO-80 (0–80)	78
	Letter fluency task 2 min (>0)	21
	Semantic fluency task 2 min (>0)	**5**
Visuospatial skills		
	Modified Taylor Complex figure (copy) (0–36)	29
Visual gnosis		
	Poppelreuter (0–8)	**4**
	PEGV (0–36)	**27**
Prefrontal functions		
	TMT A (time in s)	99
	TMT B (time in s)	223
	Stroop conflictual task (sec)	**146**
Limb praxis		
	Mahieux’s battery (0–20)	17
Visuomotor speed		
	WAIS-R coding subtest (0–133)	**26**

*in bold caps: abnormal scores.

## Conclusions

We report the case of a patient diagnosed with IPD according to the usual criteria, who presented neuropsychological impairment early in the clinical course. Her primary difficulties included impaired prefrontal functions resulting in a slight decrease in global efficiency. These abnormalities are similar, in terms of deficit and intensity, to the cognitive impairment classically observed in sporadic cases of IPD [18]. Our patient also exhibited significant impairment in visual gnosis. An alteration in visuomotor and visuoperceptive functions have been described in IPD, but the deficit is usually mild when it occurs in non-demented patients [19]. If confirmed in other patients, this unusually severe deficit in IPD could represent a red flag, alerting the clinician to perform additional diagnostic procedures.

A thorough neuropsychological assessment could prove to be useful for detecting in IPD patients who are suspected of having *C9ORF72* repeat expansions abnormalities.

## Consent

Written informed consent was obtained from the patient for publication of this case report and any accompanying images. A copy of the written consent is available for review by the Editor of this journal.

## Competing interests

The authors declare that they have no competing interests.

## Authors’ contributions

MA participated in the writing; EB took care of the patient, revised the final version of the paper; UCV took care of the patient; PV carried out the molecular genetic study; CH revised and approved the final version of the paper, KM took care of the patient, participated in the writing, revised and approved the final version.
